# Corilagin Represses Epithelial to Mesenchymal Transition Process Through Modulating Wnt/β-Catenin Signaling Cascade

**DOI:** 10.3390/biom10101406

**Published:** 2020-10-05

**Authors:** Sun Tae Hwang, Min Hee Yang, Alan Prem Kumar, Gautam Sethi, Kwang Seok Ahn

**Affiliations:** 1 Department of Science in Korean Medicine, Kyung Hee University, Seoul 02447, Korea; suntaeh12@gmail.com (S.T.H.); didmini@naver.com (M.H.Y.); 2 KHU-KIST Department of Converging Science and Technology, Kyung Hee University, Seoul 02447, Korea; 3 Department of Pharmacology, Yong Loo Lin School of Medicine, National University of Singapore, Singapore 117600, Singapore; csiapk@nus.edu.sg; 4 Cancer Science Institute of Singapore, National University of Singapore, Singapore 117599, Singapore

**Keywords:** corilagin, epithelial-to-mesenchymal transition, Wnt/β-catenin, invasion, migration

## Abstract

Corilagin (CLG), a major component of several medicinal plants, can exhibit diverse pharmacological properties including those of anti-cancer, anti-inflammatory, and hepatoprotective qualities. However, there are no prior studies on its potential impact on the epithelial-to-mesenchymal transition (EMT) process. EMT can lead to dissemination of tumor cells into other organs and promote cancer progression. Hence, we aimed to investigate the effect of CLG on EMT and its mechanism(s) of action in tumor cells. We noted that CLG reduced the expression of various epithelial markers and up-regulated the expression of Occludin and E-cadherin in both basal and TGFβ-stimulated tumor cells. CLG treatment also abrogated cellular invasion and migration in colon and prostate carcinoma cells. In addition, CLG effectively attenuated the Wnt/β-catenin signaling cascade in TGFβ-stimulated cells. Overall, our study suggests that CLG may function as and effective modulator of EMT and metastasis in neoplastic cells.

## 1. Introduction

Metastasis remains a vital hallmark by which cells originate and spread to distant institutions [1,2]. When cancer occurs, mutated cells undergo rapid proliferation in tissues of origin and then break through the basement membranes to migrate to other organs and can re-localize as a secondary metastatic cancer [3,4,5]. It is estimated that around 90% of the cancer cells in cancer patients are caused by metastasis [6,7]. These findings highlight the urgent requirement to further understand mechanisms regulating metastasis and to develop pharmacological strategies to target this process.

The epithelial-to-mesenchymal transition (EMT) can facilitate conversion of epithelial cells into mesenchymal cells and promote metastasis [8,9,10,11]. During the EMT process, the cancer cells generally have reduced the levels of epithelial markers, and display an augmentation in the levels of mesenchymal markers [12,13]. The EMT process can be triggered by various stimuli, including transforming growth factor-β (TGFβ), epidermal growth factor (EGF), and diverse signaling pathways such as wingless secreted glycoprotein (Wnt)/β-catenin, and have been implicated in cancer regulation [14,15]. Activation of the TGFβ signaling pathway is of major importance for the initiation of EMT [15,16]. TGFβ-stimulated cells can exhibit spindle-like morphology leading to a decrease of cellular polarity. In addition, the activation of TGFβ pathway can often lead to tumor progression and drug resistance [17].

A number of agents derived from Mother Nature have been reported to be efficacious against tumor growth and progression [18,19,20,21,22]. Corilagin (CLG) is one such unique component of the tannin family [23], which can be found in a wide range of medical plants such as Longan, *Lumnitzera racemose*, *Terminalia catappa* L, and *Phyllanthus* species [24,25,26]. CLG has been reported to possess many pharmacological and biological properties including anti-inflammatory, hepatoprotective, anti-microbial, antihypertensive, antidiabetic, and anti-tumor activities [27,28,29,30,31,32,33,34]. Recently, the anti-tumor effect of CLG has been focus of great interest in cancer biology [35,36,37]. It was found to attenuate cell proliferation by promoting reactive oxygen species (ROS)-dependent apoptosis and autophagy in breast and gastric cancer cells [38,39]. Jia et al. reported that CLG inhibited cell growth through TGFβ/Akt/ERK/Smad signaling pathways in ovarian cancer [36]. In addition, CLG could cause apoptosis via both the mitochondrial apoptotic pathway and death receptor pathway in hepatocellular carcinoma cells [40]. However, the actions of CLG on the regulation of EMT have not been deciphered previously, and this aspect has been studied in this report.

## 2. Material and Methods

### 2.1. Reagents

Corilagin (CLG) was procured from Sigma-Aldrich (St. Louis, MO). 3-(4,5-dimethylthiazol-2-yl)-2,5-diphenyltetrazolium bromide (MTT) and bovine serum albumin (BSA) were purchased from Sigma-Aldrich (St. Louis, MO, USA). Alexa Fluor^®^ 488 donkey anti-mouse IgG (H+L) antibody and Fluor^®^ 594 donkey anti-rabbit IgG (H+L) antibody was obtained from Life Technologies (Grand Island, NY, USA). Anti-MnSOD(sc-137254), anti-Fibronectin(sc-6952), anti-Vimentin(sc-6260), anti-E-cadherin(sc-8426), anti-N-cadherin(sc-271386), anti-Occludin(sc-5562), anti-Twist(sc-15393), anti-MMP-2(sc-53630), anti-MMP-9(sc-393859), anti-Wnt3a(sc-136163), anti-FZD-1(sc-398082), and anti-β-actin(sc-47778) antibodies were purchased from Santa Cruz Biotechnology (Santa Cruz, CA, USA). Anti-Snail(3879S), anti-Axin-1 anti-Azin-1(3323S), anti-β-catenin(9562S), anti-p-GSK3β(9322S), and anti-GSK3β(9315S) antibodies were obtained from Cell Signaling Technology (Beverly, MA, USA).

### 2.2. Cell Lines and Culture Conditions

Human colon carcinoma SNU-C2A cells, human prostate carcinoma DU145 cells, human breast carcinoma MCF-7 cells, human normal prostate RWPE-1 cells, and human normal breast MCF-10A cells were obtained from American Type Culture Collection (Manassas, VA, USA). These cells were cultured in RPMI 1640 medium containing 10% fetal bovine serum (FBS) and 1% penicillin-streptomycin. Human normal colon CCD-18Co cells were obtained from Korean Cell Line Bank (Seoul, Korea) and cells were cultured in DMEM high glucose medium containing 10% fetal bovine serum (FBS) and 1% penicillin-streptomycin.

### 2.3. MTT Assay

The viability of SNU-C2A and DU145 cells was measured using an MTT assay to detect NADH-dehydrogenase activity as elaborated before [41].

### 2.4. Western Blot Analysis

The cells were treated with CLG and TGFβ for the indicated concentrations and time points and western blotting was done as reported earlier [42]

### 2.5. Immunocytochemistry

SNU-C2A and DU145 cells were treated with CLG and TGFβ for indicated concentrations and time points. The cells were fixed with 4% paraformaldehyde at room temperature for 20 min and immunocytochemistry was done as described previously [43].

### 2.6. Real-Time Cell Proliferation Analysis

To measure cell growth, proliferation assay was performed by xCELLigence Real-Time Cell Analyzer (RTCA) DP instrument (Roche Diagnostics GmbH, Germany) as elaborated previously [44].

### 2.7. Boyden Chamber Assay

Invasion capacity of SNU-C2A and DU145 cells was determined using a 48-well micro chemotaxis Boyden chamber (Nuero Probe, Cabin John, MD, USA) as described before [45].

### 2.8. Wound Healing Assay

To measure cell migration, wound healing assay was performed. Monolayer of SNU-C2A, DU145, and PC-3 cells were scratched and treated with CLG as per the protocol described earlier [46].

### 2.9. Cell Transfection with β-Catenin siRNA

SNU-C2A and DU145 cells were transfected with β-catenin siRNA or scrambled siRNA using iN-fect^TM^ in vitro Transfection Reagent (iNtRON Biotechnology, Seongnam, KOREA) for 24 h, and thereafter, western blot analysis was carried out.

### 2.10. Statistical Analysis

The results were expressed as means ± SD, and an analysis of variance (ANOVA) with Bonferroni’s test was used for the statistical analysis.

## 3. Results

### 3.1. CLG Modulated the Expression of EMT Markers in Tumor Cells

**Figure 1:**
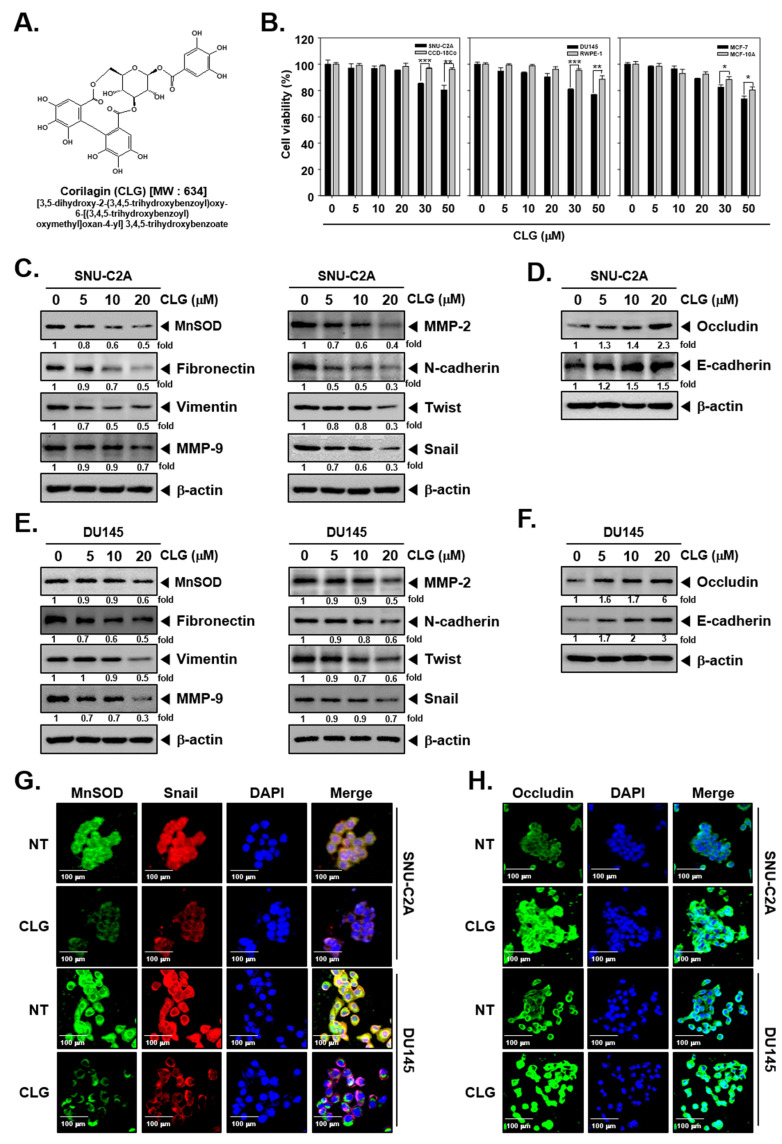
Corilagin (CLG) inhibits epithelial-mesenchymal transition (EMT) in SNU-C2A and DU145 cells. (**A**) The structure of corilagin (CLG). (**B**) Cell viability was measured by MTT assay with CLG (0, 5, 10, 20, 30, 50 μM) for 24 h. Data represent means ± SD. *p < 0.05 vs cancer cells, **p < 0.01 vs cancer cells, ***p < 0.001 vs cancer cells. (**C**–**F**) SNU-C2A and DU145 cells were exposed to CLG for 24 h. Whole cell lysate were extracted levels of various proteins was evaluated by Western blot analysis. (**G**) and (**H**) Expression of MnSOD, Snail, and Occludin was measured by immunocytochemistry in SNU-C2A and DU145 cells.

The structure of CLG has been depicted in Figure 1A. The cytotoxic action of CLG in SNU-C2A, CCD-18Co, DU145, RWPE-1, MCF-7, and MCF-10A cells was first determined. Interestingly, CLG did not exhibit any marked effect on cell viability in human colon, prostate, breast carcinoma, and normal epithelial cells. The results demonstrated that the cytotoxicity of CLG was less than 10% at 20 μM concentration (Figure 1B). Hence, in vitro experiments were carried out below the 20 μM dose. We also determined whether CLG can modulate the levels of EMT markers. Interestingly, the levels of MnSOD, Fibronectin, Vimentin, MMP-9, MMP-2, N-cadherin, Twist, and Snail was suppressed by CLG. However, the levels of Occludin and E-cadherin were increased (Figure 1C–F). Immunohistochemical analysis as shown in Figure 1G,H, also revealed that the expression of MnSOD and Snail was down-regulated, whereas expression of Occludin was increased in SNU-C2A and DU145 cells.

### 3.2. CLG Effectively Attenuated Cellular Invasion and Migration

**Figure 2:**
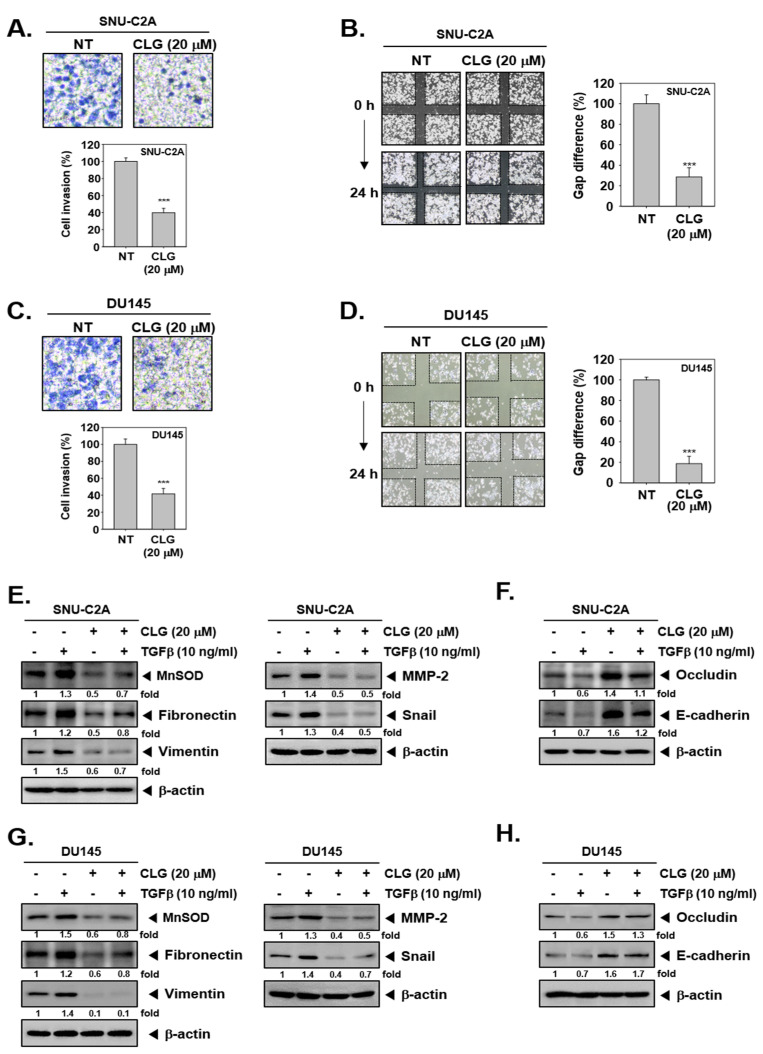
CLG suppressed invasion as well as migration. SNU-C2A and DU145 cells were exposed to CLG (20 μM) for 24 h. (**A** and **C**) Invasive activity was measured by Boyden chamber assay in SNU-C2A and DU45 cells. Data represent means ± SD. ***p < 0.001. (**B** and **D**) The cells were scratched and wound healing assay for evaluate cell migration. Data represent means ± SD. ***p < 0.001. (**E**–**H**) SNU-C2A and DU145 cells were treated with TGFβ (10 ng/mL), CLG (20 μM), or a combination for 24 h. Whole cell lysates were extracted and expression of EMT-related proteins was analyzed by western blotting. The results shown are representative of the three independent experiments.

Invasive activity of SNU-C2A and DU145 cells was investigated by Boyden chamber assay. As shown in Figure 2A,C, CLG was observed to attenuate invasion of cells.

In addition, migration was examined by wound healing assay. The results showed that CLG significantly reduced migratory ability in the treated cells (Figure 2B,D).

### 3.3. CLG Abrogated TGFβ-Induced EMT Cascade

**Figure 3:**
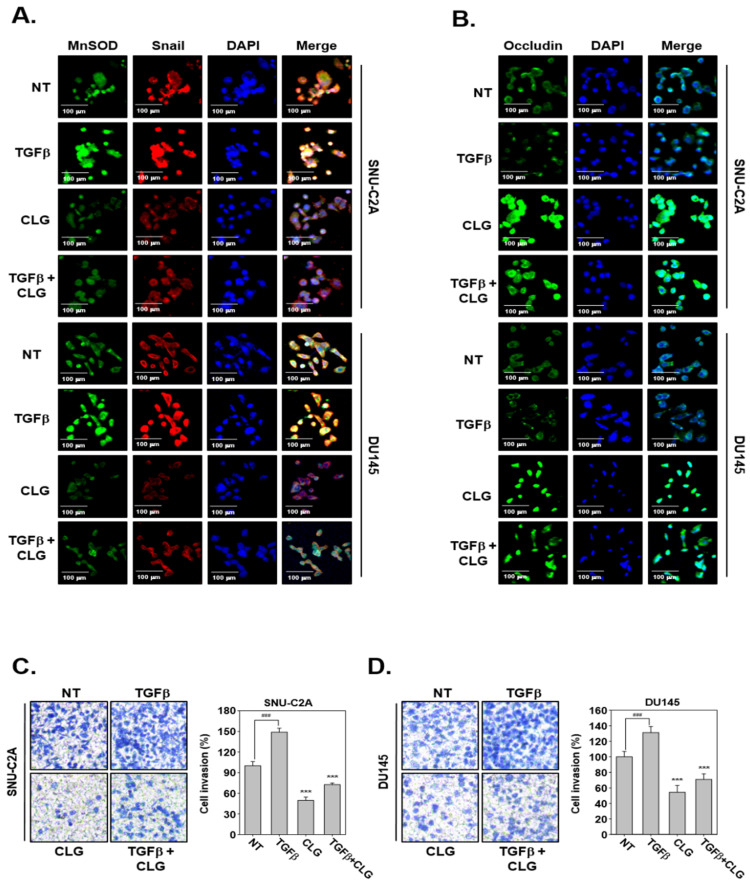
CLG reduced TGFβ-induced EMT process and cell invasion. SNU-C2A and DU145 cells were exposed to TGFβ (10 ng/mL), CLG (20 μM), or a combination for 24 h. (**A** and **B**) Expression of MnSOD as well as occludin was observed in green, and Snail was observed in red by immunocytochemistry. (**C** and **D**) Invasion assay with Boyden chamber was performed. Data represent means ± SD. ^###^*p* < 0.001 vs. non-treated (NT) cells and ***p < 0.001 vs TGFβ-treated cells.

Next, we determined whether CLG can also modulate TGFβ-driven EMT in SNU-C2A and DU145 cells. Western blotting data revealed that exposure to TGFβ up-regulated levels of various proteins regulating EMT and invasion processes. Interestingly, CLG treatment reversed TGFβ-induced up-regulation of these oncogenic proteins (Figure 2E,G). Moreover, TGFβ down-regulated occludin and E-cadherin expression and CLG treatment could reverse these changes effectively (Figure 2F,H). In addition, immunocytochemistry data showed that, CLZ suppressed TGFβ-induced MnSOD and Snail levels, whereas it enhanced occludin levels (Figure 3A,B).

### 3.4. CLG Suppressed TGFβ-Induced Metastatic Effects

**Figure 4:**
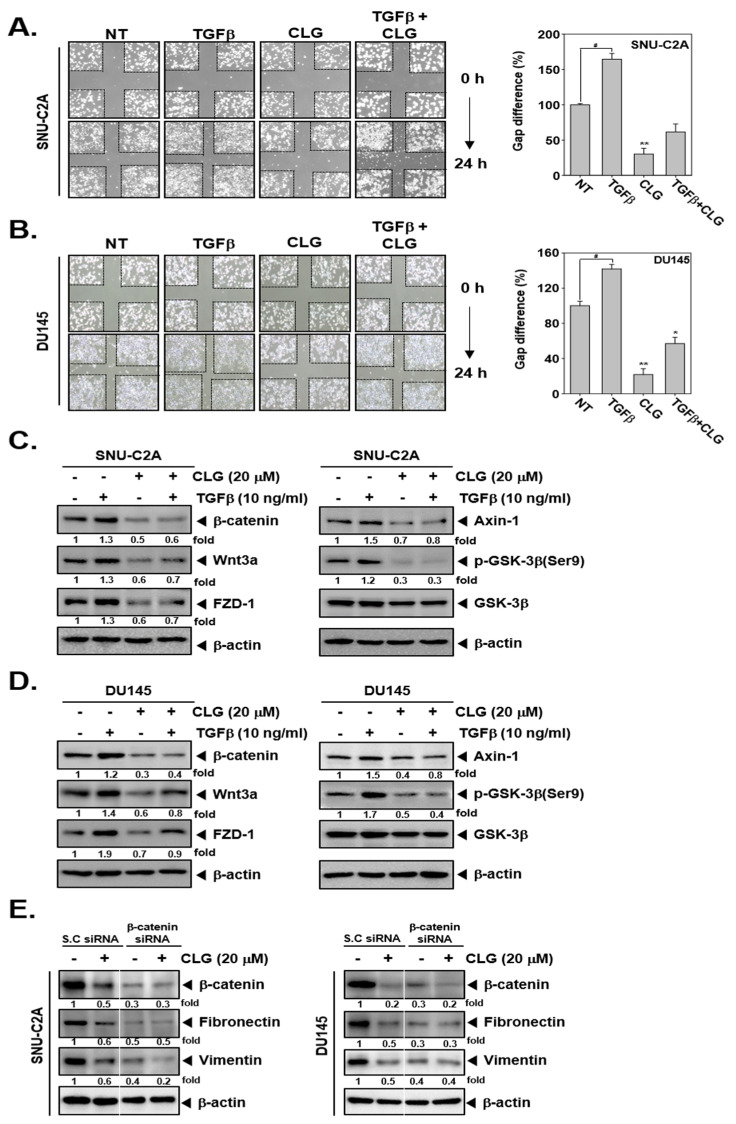
CLG down-regulated Wnt/β-catenin signaling cascades. SNU-C2A and DU145 cells were exposed to TGFβ (10 ng/mL), CLG (20 μM), or a combination for 24 h. (**A** and **B**) Cell migration was determined by wound healing assay. Data represent means ± SD. #*p* < 0.05 vs non-treated (NT) cells, *p < 0.05 vs TGFβ treated cells, and **p < 0.01 vs TGFβ treated cells. (**C** and **D**) Western blotting for Wnt/β-catenin signaling cascades such as β-catenin, Wnt3a, FZD-1, Axin-1, p-GSK-3β, and GSK-3β. β-actin was used as a loading control. The results shown are representative of the three independent experiments. (**E**) SNU-C2A and DU145 cells were transfected with β-catenin siRNA or scrambled siRNA (100 nM) for 24 h. After that, cells were treated with CLG for 24 h and Western blotting for various antibodies was done.

It was also noted that CLG suppressed TGFβ-induced cellular invasion in SNU-CA and DU145 cells (Figure 3C,D). TGFβ-treated cells displayed enhanced invasive property, whereas CLG treatment prevented TGFβ-induced invasion. In addition, wound healing assay data revealed that TGFβ induced migration, but CLG suppressed this activity significantly (Figure 4A,B).

### 3.5. CLG Down-Regulated TGFβ-Induced Activation of Wnt/β-Catenin Pathway

We investigated whether CLG could affect the Wnt/β-catenin signaling pathway in SNU-C2A and DU145 cells. As shown in Figure 4C,D, CLG substantially suppressed TGF β-induced the levels of β-catenin, Wnt3a, FZD-1, Axin-1, and GSK-3β activation. We also performed a β-catenin knockdown study to confirm the relationship between CLG-induced modulation of Wnt/β-catenin pathway and EMT proteins. As shown in Figure 4E, β-catenin, Fibronectin, and Vimentin expression was suppressed in β-catenin siRNA transfected cells. However, the expression of these proteins was not altered substantially in the cells transfected with scrambled siRNA, thus indicating that β-catenin may act as a potential molecular target primarily affected by CLG.

## 4. Discussions

Corilagin (CLG) can be isolated from wide range of medical plants [23,24] and can display many biological activities including those of anti-inflammatory, hepatoprotective, anti-microbial, antihypertensive, antidiabetic, and anti-tumor [27,28,29,30,31,32,33,34], but its impact on EMT has not been well characterized yet. We report here for the first time that CLG reduced levels of different mesenchymal markers and enhanced that of epithelial markers (occludin and E-cadherin) in both SNU-C2A and DU145 cells. In addition, we found that CLG abrogated TGFβ-induced expression of various proteins regulating EMT, whereas it increased TGFβ-promoted reduction in occludin and N-cadherin levels. Interestingly, CLG-induced alteration of EMT was observed to be associated with attenuation of invasion and migration. In addition, CLG mitigated constitutive as well as TGFβ-induced Wnt/β-catenin signaling pathway activation in tumor cells.

EMT can alter the levels of different epithelial markers and then cause transformation into mesenchymal state via up-regulating the levels of important markers such as Fibronectin, Vimentin, N-cadherin [47,48]. Consequently, these mesenchymal cells can exhibit enhanced motility and have increased ability to undergo metastasis [49]. In addition, previous reports have found that polyphenol and resveratrol can effectively suppress cell migration and invasion in human prostate and colorectal carcinoma cells [50,51]. Moreover, resveratrol also modulated the expression of EMT-related markers such as E-cadherin and Vimentin in DU145 and PC3 cells [50]. We observed that CLG down-regulated MnSOD, fibronectin, Vimentin, and N-cadherin in SNU-C2A and DU145 cells. The levels of occludin and E-cadherin were also effectively up-regulated by CLG in these cells. In addition, CLG down-regulated the levels of E-cadherin repressor proteins, Twist and Snail. Matrix metalloproteinases (MMP) family such as MMP-9 and MMP-2 can play an important role in cancer proliferation, invasion, and metastasis [52,53,54]. We observed that CLG inhibited the level of both MMP-9 and MMP-2 proteins, thus implicating that the negative regulation of invasion and metastasis by CLG may be mediated by down-regulation of these two proteins. Overall, alteration of diverse mesenchymal markers and epithelial markers by CLG can abrogate EMT in tumor cell lines.

TGFβ can regulate EMT process and tumorigenesis, thus leading to enhanced motility and invasion [55]. Previous studies have reported that mesenchymal markers were found to be substantially overexpressed and epithelial markers were suppressed in TGFβ-stimulated cells [8,43,44]. We also noted that CLG suppressed the levels of up-regulated EMT-associated proteins and down-regulated E-cadherin and occludin expression in TGFβ-induced SNU-C2A and DU-145 cells. We further observed that CLG altered the morphological transformation as well as migration and invasion induced by TGFβ. Our findings suggest that CLG can effectively target TGFβ-induced EMT and subsequent downstream phenotypic changes in tumor cells.

A number of cellular signaling pathways can regulate EMT, such as HGF, EGF, TGFβ, Notch, Wnt/β-catenin, etc. [56,57]. Activation of the Wnt/β-catenin signaling cascade is common in various malignancies including colon and prostate cancers [58,59,60,61]. The Wnt/β-catenin can act as an important regulator of EMT in many different types of cancers [62,63,64,65,66]. Wnt/β-catenin signaling pathway has been reported to cooperate with TGβ signaling in the orchestration of the EMT response [15]. Bernaudo et al. reported that cyclin G2 potently suppressed EMT through inhibition of the Wnt/β-catenin signaling pathway by down-regulating LRP6, DVL2, and β-catenin [64]. In addition, aspirin can suppress colon cancer migration through modulating EMT by Wnt signaling [67]. In this study, we noted that CLG diminished TGFβ-promoted activation of Wnt/β-catenin pathway in both SNU-C2A and DU145 cells. CLG suppressed TGFβ-induced activation of various proteins such as β-catenin, Wnt3a, FZD-1, Axin-1, and GSK-3β substantially. We also noted that the inhibitory actions of CLG on EMT-related proteins were predominantly mediated through Wnt/β-catenin signaling pathway based on the findings of our siRNA experiments.

Overall, our findings suggest that the potential effects of CLG on constitutive and TGFβ-induced EMT process through modulating Wnt/β-catenin signaling pathway. We noticed that CLG attenuated the levels of mesenchymal markers and up-regulated that of epithelial markers. In addition, CLG inhibited cell invasion and metastasis by causing blockage of EMT driven signals. Moreover, CLG abrogated Wnt/β-catenin signaling cascade in TGFβ-stimulated tumor cells. However, additional experiments are required to evaluate the anti-metastatic potential of CLG in suitable preclinical models.

## References

[1] Bacac M., Stamenkovic I. (2008). Metastatic cancer cell. Ann. Rev. Pathol..

[2] Zhang J., Ahn K.S., Kim C., Shanmugam M.K., Siveen K.S., Arfuso F., Samym R.P., Deivasigamanim A., Lim L.H., Wang L. (2016). Nimbolide-Induced Oxidative Stress Abrogates STAT3 Signaling Cascade and Inhibits Tumor Growth in Transgenic Adenocarcinoma of Mouse Prostate Model. Antioxid. Redox Signal..

[3] Fidler I.J. (1989). Origin and biology of cancer metastasis. Cytometry.

[4] Wee I., Syn N., Sethi G., Goh B.C., Wang L. (2019). Role of tumor-derived exosomes in cancer metastasis. Biochim. Biophys. Acta. Rev. Cancer.

[5] Shanmugam M.K., Manu K.A., Ong T.H., Ramachandran L., Surana R., Bist P., Lim L.H., Kumar A.P., Hui K.M., Sethi G. (2011). Inhibition of CXCR4/CXCL12 signaling axis by ursolic acid leads to suppression of metastasis in transgenic adenocarcinoma of mouse prostate model. Int. J. Cancer.

[6] Mehlen P., Puisieux A. (2006). Metastasis: A question of life or death. Nat. Rev. Cancer.

[7] Chua A.W., Hay H.S., Rajendran P., Shanmugam M.K., Li F., Bist P., Koay E.S., Lim L.H., Kumar A.P., Sethi G. (2010). Butein downregulates chemokine receptor CXCR4 expression and function through suppression of NF-kappaB activation in breast and pancreatic tumor cells. Biochem. Pharmacol..

[8] Ko J.H., Nam D., Um J.Y., Jung S.H., Sethi G., Ahn K.S. (2018). Bergamottin Suppresses Metastasis of Lung Cancer Cells through Abrogation of Diverse Oncogenic Signaling Cascades and Epithelial-to-Mesenchymal Transition. Molecules.

[9] Nieszporek A., Skrzypek K., Adamek G., Majka M. (2019). Molecular mechanisms of epithelial to mesenchymal transition in tumor metastasis. Acta Biochim. Pol..

[10] Loh C.Y., Chai J.Y., Tang T.F., Wong W.F., Sethi G., Shanmugam M.K., Chong P.P., Looi C.Y. (2019). The E-Cadherin and N-Cadherin Switch in Epithelial-to-Mesenchymal Transition: Signaling, Therapeutic Implications, and Challenges. Cells.

[11] Cheng J.T., Wang L., Wang H., Tang F.R., Cai W.Q., Sethi G., Xin H.W., Ma Z. (2019). Insights into Biological Role of LncRNAs in Epithelial-Mesenchymal Transition. Cells.

[12] Krantz S.B., Shields M.A., Dangi-Garimella S., Munshi H.G., Bentrem D.J. (2012). Contribution of epithelial-to-mesenchymal transition and cancer stem cells to pancreatic cancer progression. J. Surg. Res..

[13] Lee J.H., Chinnathambi A., Alharbi S.A., Shair O.H.M., Sethi G., Ahn K.S. (2019). Farnesol abrogates epithelial to mesenchymal transition process through regulating Akt/mTOR pathway. Pharmacol. Res..

[14] Nelson W.J., Nusse R. (2004). Convergence of Wnt, beta-catenin, and cadherin pathways. Science.

[15] Xu J., Lamouille S., Derynck R. (2009). TGF-beta-induced epithelial to mesenchymal transition. Cell Res..

[16] Ioannou M., Kouvaras E., Papamichali R., Samara M., Chiotoglou I., Koukoulis G. (2018). Smad4 and epithelial-mesenchymal transition proteins in colorectal carcinoma: An immunohistochemical study. J. Mol. Histol..

[17] Zhu H., Gu X., Xia L., Zhou Y., Bouamar H., Yang J., Ding X., Zwieb C., Zhang J., Hinck A.P. (2018). A Novel TGFbeta Trap Blocks Chemotherapeutics-Induced TGFbeta1 Signaling and Enhances Their Anticancer Activity in Gynecologic Cancers. Clin. Cancer Res. Off. J. Am. Assoc. Cancer Res..

[18] Kim C., Cho S.K., Kapoor S., Kumar A., Vali S., Abbasi T., Kim S.H., Sethi G., Ahn K.S. (2014). β-Caryophyllene oxide inhibits constitutive and inducible STAT3 signaling pathway through induction of the SHP-1 protein tyrosine phosphatase. Mol. Carcinog..

[19] Siveen K.S., Ahn K.S., Ong T.H., Shanmugam M.K., Li F., Yap W.N., Kumar A.P., Fong C.W., Tergaonkar V., Hui K.M. (2014). γ-tocotrienol inhibits angiogenesis-dependent growth of human hepatocellular carcinoma through abrogation of AKT/mTOR pathway in an orthotopic mouse model. Oncotarget.

[20] Siveen K.S., Mustafa N., Li F., Kannaiyan R., Ahn K.S., Kumar A.P., Chng W.J., Sethi G. (2014). Thymoquinone overcomes chemoresistance and enhances the anticancer effects of bortezomib through abrogation of NF-kappaB regulated gene products in multiple myeloma xenograft mouse model. Oncotarget.

[21] Shanmugam M.K., Warrier S., Kumar A.P., Sethi G., Arfuso F. (2017). Potential Role of Natural Compounds as Anti-Angiogenic Agents in Cancer. Curr. Vasc. Pharmacol..

[22] Dai X., Zhang J., Arfuso F., Chinnathambi A., Zayed M.E., Alharbi S.A., Kumar A.P., Ahn K.S., Sethi G. (2015). Targeting TNF-related apoptosis-inducing ligand (TRAIL) receptor by natural products as a potential therapeutic approach for cancer therapy. Exp. Biol. Med..

[23] Gupta A., Singh A.K., Kumar R., Ganguly R., Rana H.K., Pandey P.K., Sethi G., Bishayee A., Pandey A.K. (2019). Corilagin in Cancer: A Critical Evaluation of Anticancer Activities and Molecular Mechanisms. Molecules.

[24] Rangkadilok N., Worasuttayangkurn L., Bennett R.N., Satayavivad J. (2005). Identification and quantification of polyphenolic compounds in Longan (*Euphoria longana* Lam.) fruit. J. Agric. Food Chem..

[25] Darwish A.G., Samy M.N., Sugimoto S., Otsuka H., Abdel-Salam H., Matsunami K. (2016). Effects of Hepatoprotective Compounds from the Leaves of Lumnitzera racemosa on Acetaminophen-Induced Liver Damage in Vitro. Chem. Pharm. Bull..

[26] Notka F., Meier G.R., Wagner R. (2003). Inhibition of wild-type human immunodeficiency virus and reverse transcriptase inhibitor-resistant variants by *Phyllanthus amarus*.. Antivir. Res..

[27] Tang Y.Y., He X.M., Sun J., Li C.B., Li L., Sheng J.F., Xin M., Li Z.C., Zheng F.J., Liu G.M. (2019). Polyphenols and Alkaloids in Byproducts of Longan Fruits (Dimocarpus Longan Lour.) and Their Bioactivities. Molecules.

[28] Kolodziej H., Burmeister A., Trun W., Radtke O.A., Kiderlen A.F., Ito H., Hatano T., Yoshida T., Foo L.Y. (2005). Tannins and related compounds induce nitric oxide synthase and cytokines gene expressions in Leishmania major-infected macrophage-like RAW 264.7 cells. Bioorg. Med. Chem..

[29] Jin F., Cheng D., Tao J.Y., Zhang S.L., Pang R., Guo Y.J., Ye P., Dong J.H., Zhao L. (2013). Anti-inflammatory and anti-oxidative effects of corilagin in a rat model of acute cholestasis. BMC Gastroenterol..

[30] Miyasaki Y., Rabenstein J.D., Rhea J., Crouch M.L., Mocek U.M., Kittell P.E., Morgan M.A., Nichols W.S., Van Benschoten M.M., Hardy W.D. (2013). Isolation and characterization of antimicrobial compounds in plant extracts against multidrug-resistant Acinetobacter baumannii. PLoS ONE.

[31] Cheng J.T., Lin T.C., Hsu F.L. (1995). Antihypertensive effect of corilagin in the rat. Can. J. Physiol. Pharmacol..

[32] Yang M.H., Vasquez Y., Ali Z., Khan I.A., Khan S.I. (2013). Constituents from Terminalia species increase PPARalpha and PPARgamma levels and stimulate glucose uptake without enhancing adipocyte differentiation. J. Ethnopharmacol..

[33] Lipinska L., Klewicka E., Sojka M. (2014). The structure, occurrence and biological activity of ellagitannins: A general review. Acta Sci. Polonorum. Technol. Aliment..

[34] Zheng Z.Z., Chen L.H., Liu S.S., Deng Y., Zheng G.H., Gu Y., Ming Y.L. (2016). Bioguided Fraction and Isolation of the Antitumor Components from Phyllanthus niruri L.. Biomed Res. Int..

[35] Ming Y., Zheng Z., Chen L., Zheng G., Liu S., Yu Y., Tong Q. (2013). Corilagin inhibits hepatocellular carcinoma cell proliferation by inducing G2/M phase arrest. Cell Biol. Int..

[36] Jia L., Jin H., Zhou J., Chen L., Lu Y., Ming Y., Yu Y. (2013). A potential anti-tumor herbal medicine, Corilagin, inhibits ovarian cancer cell growth through blocking the TGF-beta signaling pathways. BMC Complement. Altern. Med..

[37] Gambari R., Hau D.K., Wong W.Y., Chui C.H. (2014). Sensitization of Hep3B hepatoma cells to cisplatin and doxorubicin by corilagin. Phytother. Res..

[38] Tong Y., Zhang G., Li Y., Xu J., Yuan J., Zhang B., Hu T., Song G. (2018). Corilagin inhibits breast cancer growth via reactive oxygen species-dependent apoptosis and autophagy. J. Cell. Mol. Med..

[39] Xu J., Zhang G., Tong Y., Yuan J., Li Y., Song G. (2019). Corilagin induces apoptosis, autophagy and ROS generation in gastric cancer cells in vitro. Int. J. Mol. Med..

[40] Deng Y., Li X., Li X., Zheng Z., Huang W., Chen L., Tong Q., Ming Y. (2018). Corilagin induces the apoptosis of hepatocellular carcinoma cells through the mitochondrial apoptotic and death receptor pathways. Oncol. Rep..

[41] Hwang S.T., Um J.Y., Chinnathambi A., Alharbi S.A., Narula A.S., Namjoshi O.A., Blough B.E., Ahn K.S. (2020). Evodiamine Mitigates Cellular Growth and Promotes Apoptosis by Targeting the c-Met Pathway in Prostate Cancer Cells. Molecules.

[42] Yang M.H., Jung S.H., Chinnathambi A., Alahmadi T.A., Alharbi S.A., Sethi G., Ahn K.S. (2019). Attenuation of STAT3 Signaling Cascade by Daidzin Can Enhance the Apoptotic Potential of Bortezomib against Multiple Myeloma. Biomolecules.

[43] Yang M.H., Lee J.H., Ko J.H., Jung S.H., Sethi G., Ahn K.S. (2019). Brassinin Represses Invasive Potential of Lung Carcinoma Cells through Deactivation of PI3K/Akt/mTOR Signaling Cascade. Molecules.

[44] Baek S.H., Ko J.H., Lee J.H., Kim C., Lee H., Nam D., Lee J., Lee S.G., Yang W.M., Um J.Y. (2017). Ginkgolic Acid Inhibits Invasion and Migration and TGF-beta-Induced EMT of Lung Cancer Cells Through PI3K/Akt/mTOR Inactivation. J. Cell. Physiol..

[45] Kim S.M., Oh E.Y., Lee J.H., Nam D., Lee S.G., Lee J., Kim S.H., Shim B.S., Ahn K.S. (2015). Brassinin Combined with Capsaicin Enhances Apoptotic and Anti-metastatic Effects in PC-3 Human Prostate Cancer Cells. Phytother. Res..

[46] Baek S.H., Ko J.H., Lee H., Jung J., Kong M., Lee J.W., Lee J., Chinnathambi A., Zayed M.E., Alharbi S.A. (2016). Resveratrol inhibits STAT3 signaling pathway through the induction of SOCS-1: Role in apoptosis induction and radiosensitization in head and neck tumor cells. Phytomed. Int. J. Phytother. Phytopharm..

[47] Kim Y.N., Koo K.H., Sung J.Y., Yun U.J., Kim H. (2012). Anoikis resistance: An essential prerequisite for tumor metastasis. Int. J. Cell Biol..

[48] Lee J.M., Dedhar S., Kalluri R., Thompson E.W. (2006). The epithelial-mesenchymal transition: New insights in signaling, development, and disease. J. Cell Biol..

[49] Lee H., Ko J.H., Baek S.H., Nam D., Lee S.G., Lee J., Yang W.M., Um J.Y., Kim S.H., Shim B.S. (2016). Embelin Inhibits Invasion and Migration of MDA-MB-231 Breast Cancer Cells by Suppression of CXC Chemokine Receptor 4, Matrix Metalloproteinases-9/2, and Epithelial-Mesenchymal Transition. Phytother. Res..

[50] Khusbu F.Y., Zhou X., Roy M., Chen F.Z., Cao Q., Chen H.C. (2020). Resveratrol induces depletion of TRAF6 and suppresses prostate cancer cell proliferation and migration. Int. J. Biochem. Cell Biol..

[51] Yuan L., Zhou M., Huang D., Wasan H.S., Zhang K., Sun L., Huang H., Ma S., Shen M., Ruan S. (2019). Resveratrol inhibits the invasion and metastasis of colon cancer through reversal of epithelial mesenchymal transition via the AKT/GSK3beta/Snail signaling pathway. Mol. Med. Rep..

[52] Sani I.K., Marashi S.H., Kalalinia F. (2015). Solamargine inhibits migration and invasion of human hepatocellular carcinoma cells through down-regulation of matrix metalloproteinases 2 and 9 expression and activity. Toxicol. Vitr. Int. J. Publ. Assoc. Bibra.

[53] Shay G., Lynch C.C., Fingleton B. (2015). Moving targets: Emerging roles for MMPs in cancer progression and metastasis. Matrix Biol. J. Int. Soc. Matrix Biol..

[54] Ahn K.S., Sethi G., Jain A.K., Jaiswal A.K., Aggarwal B.B. (2006). Genetic deletion of NAD(P)H:quinone oxidoreductase 1 abrogates activation of nuclear factor-kappaB, IkappaBalpha kinase, c-Jun N-terminal kinase, Akt, p38, and p44/42 mitogen-activated protein kinases and potentiates apoptosis. J. Biol. Chem..

[55] Massague J. (2008). TGFbeta in Cancer. Cell.

[56] Polyak K., Weinberg R.A. (2009). Transitions between epithelial and mesenchymal states: Acquisition of malignant and stem cell traits. Nat. Rev. Cancer.

[57] Bhuvanalakshmi G., Basappa, Rangappa K.S., Dharmarajan A., Sethi G., Kumar A.P., Warrier S. (2017). Breast Cancer Stem-Like Cells Are Inhibited by Diosgenin, a Steroidal Saponin, by the Attenuation of the Wnt beta-Catenin Signaling via the Wnt Antagonist Secreted Frizzled Related Protein-4. Front. Pharmacol..

[58] Huang J., Xiao D., Li G., Ma J., Chen P., Yuan W., Hou F., Ge J., Zhong M., Tang Y. (2014). EphA2 promotes epithelial-mesenchymal transition through the Wnt/beta-catenin pathway in gastric cancer cells. Oncogene.

[59] Nagaraj A.B., Joseph P., Kovalenko O., Singh S., Armstrong A., Redline R., Resnick K., Zanotti K., Waggoner S., DiFeo A. (2015). Critical role of Wnt/beta-catenin signaling in driving epithelial ovarian cancer platinum resistance. Oncotarget.

[60] Yue B., Liu C., Sun H., Liu M., Song C., Cui R., Qiu S., Zhong M. (2018). A Positive Feed-Forward Loop between LncRNA-CYTOR and Wnt/beta-Catenin Signaling Promotes Metastasis of Colon Cancer. Mol. Ther. J. Am. Soc. Gene Ther..

[61] Murillo-Garzon V., Kypta R. (2017). WNT signalling in prostate cancer. Nat. Rev. Urol..

[62] Wu Z.Q., Li X.Y., Hu C.Y., Ford M., Kleer C.G., Weiss S.J. (2012). Canonical Wnt signaling regulates Slug activity and links epithelial-mesenchymal transition with epigenetic Breast Cancer 1, Early Onset (BRCA1) repression. Proc. Natl. Acad. Sci. USA.

[63] Yook J.I., Li X.Y., Ota I., Fearon E.R., Weiss S.J. (2005). Wnt-dependent regulation of the E-cadherin repressor snail. J. Biol. Chem..

[64] Bernaudo S., Salem M., Qi X., Zhou W., Zhang C., Yang W., Rosman D., Deng Z., Ye G., Yang B.B. (2016). Cyclin G2 inhibits epithelial-to-mesenchymal transition by disrupting Wnt/beta-catenin signaling. Oncogene.

[65] Bhuvanalakshmi G., Gamit N., Patil M., Arfuso F., Sethi G., Dharmarajan A., Kumar A.P., Warrier S. (2018). Stemness, Pluripotentiality and Wnt Antagonism: sFRP4, a Wnt antagonist Mediates Pluripotency and Stemness in Glioblastoma. Cancers.

[66] Ong M.S., Cai W., Yuan Y., Leong H.C., Tan T.Z., Mohammad A., You M.L., Arfuso F., Goh B.C., Warrier S. (2017). ‘Lnc’-ing Wnt in female reproductive cancers: Therapeutic potential of long non-coding RNAs in Wnt signalling. Br. J. Pharmacol..

[67] Jin S., Wu X. (2019). Aspirin inhibits colon cancer cell line migration through regulating epithelial-mesenchymal transition via Wnt signaling. Oncol. Lett..

